# Rice putative methyltransferase gene *OsTSD2* is required for root development involving pectin modification

**DOI:** 10.1093/jxb/erw297

**Published:** 2016-08-06

**Authors:** Lianghuan Qu, Chunyan Wu, Fei Zhang, Yangyang Wu, Chuanying Fang, Cheng Jin, Xianqing Liu, Jie Luo

**Affiliations:** ^1^National Key Laboratory of Crop Genetic Improvement and National Center of Plant Gene Research (Wuhan), Huazhong Agricultural University, Wuhan 430070, China; ^2^College of Life Science and Technology, Huazhong Agricultural University, Wuhan 430070, China

**Keywords:** Aerenchyma, cellular adhesion, methyltransferase, *Oryza sativa*, pectin, root development.

## Abstract

Pectin synthesis and modification are vital for plant development, although the underlying mechanisms are still not well understood. Here, we report the functional characterization of the *OsTSD2* gene, which encodes a putative methyltransferase in rice. All three independent T-DNA insertion lines of *OsTSD2* displayed dwarf phenotypes and serial alterations in different zones of the root. These alterations included abnormal cellular adhesion and schizogenous aerenchyma formation in the meristematic zone, inhibited root elongation in the elongation zone, and higher lateral root density in the mature zone. Immunofluorescence (with LM19) and Ruthenium Red staining of the roots showed that unesterified homogalacturonan (HG) was increased in *Ostsd2* mutants. Biochemical analysis of cell wall pectin polysaccharides revealed that both the monosaccharide composition and the uronic acid content were decreased in *Ostsd2* mutants. Increased endogenous ABA content and opposite roles performed by ABA and IAA in regulating cellular adhesion in the *Ostsd2* mutants suggested that *OsTSD2* is required for root development in rice through a pathway involving pectin synthesis/modification. A hypothesis to explain the relationship among *OsTSD2*, pectin methylesterification, and root development is proposed, based on pectin’s function in regional cell extension/division in a zone-dependent manner.

## Highlight

The rice putative methyltransferase gene *OsTSD2* is functionally identified and is shown to control root development in a way that involves pectin synthesis and modification in a zone-dependent manner.

## Introduction

Pectin is structurally and functionally the most complex polysaccharide in plant cell walls (Mohnen, 2008). Multiple lines of evidence indicate that pectin plays essential roles in plant growth and development, which is consistent with the retention of a large number of genes required for pectin synthesis and modification (Ridley *et al.*, 2001; Mohnen, 2008; Atmodjo *et al.*, 2013). Biochemically, pectins are a group of polysaccharides that are rich in galacturonic acid (GalA) and can be classified into three main types: homogalacturonan (HG), rhamnogalacturonan-I (RG-I), and rhamnogalacturonan-II (RG- II) (Willats *et al.*, 2001). HG biosynthesis and modification have recently emerged as key determinants of plant development (Wolf *et al.*, 2009).

As an abundant and widespread type of pectin, HG is a linear homopolymer of (1→4)-α-linked-D-galacturonic acid molecules (Willats *et al.*, 2001). HG appears to be synthesized in the Golgi apparatus and is deposited in the cell wall in a form in which 70–80% of GalA residues are methylesterified at the C-6 carboxyl group (O’Neill *et al*., 1990). The removal of methyl ester groups within the cell wall matrix makes HG capable of being cross-linked by calcium and also makes the formation of supramolecular assemblies and gels possible (Willats *et al.*, 2001). It is generally accepted that the amount and distribution of methyl groups affect the pectin matrix’s rheological properties, adhesive capacities, and resistance to degradation (Wolf *et al.*, 2009, 2012). These changes then affect plant growth and development through multiple processes, including organ initiation (Peaucelle *et al.*, 2011), the maintenance of the stem’s mechanical strength (Hongo *et al.*, 2012), and pollen formation (Francis *et al.*, 2006). Immunological and spectroscopic detection of highly methyl- and demethylesterified HG epitopes suggests that the former are associated with extensible walls in growing parts of the cell, whereas the latter are associated with non-extensible walls (Wolf *et al.*, 2012). For example, in the Arabidopsis root tip, the walls of non-dividing cells (i.e. the central cells of the quiescent center) contain higher levels of unesterified pectin compared with dividing (meristematic) cells (Dolan *et al.*, 1997). By observing calcium distribution and pectin esterification patterns in the cambial zones of poplar branches, the degree of HG methylesterification has been determined to vary from one cell type to another (Guglielmino *et al.*, 1997). These reports indicate that the degree of pectin methylesterification in plants is under subtle regulation to maintain proper organogenesis and normal morphogenesis. Enzymatic activities that regulate the degree of HG methylesterification are likely to play major roles in the control of plant growth (Wolf *et al.*, 2009).

There are two kinds of closely related enzymes that regulate the degree of HG methylesterification: homogalacturonan methyltransferases (HG-MTs), which are usually located in the Golgi apparatus and catalyze the transfer of a methyl group (CH_3_) from the donor substrate S-adenosyl-L-methionine (SAM) to the C-6 carboxyl group of α-1,4-linked GalA residues in HG acceptors (Atmodjo *et al.*, 2013), and pectic methylesterases (PMEs), which are usually cell wall-localized and can remove the methyl groups from HG (Wolf *et al.*, 2009). Because HG is usually secreted from the Golgi apparatus in a highly methylesterified form and is then de-esterified by PMEs, it is well accepted that the methylation status of HG is mainly controlled by cell wall-localized esterases (Wolf *et al.*, 2009). However, in the cambial zones of poplar branches, significant labeling with the JIM5 antibody (which recognizes low-esterified HG) has been observed in some dictyosomes and vesicles, and might reflect the exocytosis of low-methylesterified material in PME-lacking domains (Guglielmino *et al.*, 1997). This finding suggests that in addition to the main secretory pathway for methylesterified pectin, there might be an alternative secondary pathway for acidic pectin (Roberts, 1990).

HG-MT activity was first detected in particulate fractions of the mung bean, *Phaseolus aureus* (Kauss and Hassid, 1967). Since then, Golgi-localized HG-MT activity has been reported in *Linum usitatissimum* (Vannier *et al.*, 1992), *Nicotiana tabacum* (Goubet *et al.*, 1998; Goubet and Mohnen, 1999), *Glycine max* (Ishikawa *et al.*, 2000), and *Pisum sativum* (Ibar and Orellana, 2007). Several putative HG-MTs have also been described in Arabidopsis, including AtQUA2, AtQUA3, and AtCGR3. Although none of these putative HG-MTs have been characterized biochemically, mutation of the genes that encode them can induce alterations in pectin components and the degree of HG methylesterification, and can even cause deficiencies in plant development (Mouille *et al.*, 2007; Held *et al.*, 2011; Miao *et al.*, 2011; Atmodjo *et al.*, 2013). For example, *Atqua2*/*Attsd2* mutants are dwarfed and exhibit reduced cell adhesion due to altered cell wall composition (Mouille *et al.*, 2007). Functional analysis of *AtQUA2*/*AtTSD2* showed that HG-MT may be pleiotropic because it is involved in co-ordinating plant shoot development (Frank *et al.*, 2002; Krupková *et al.*, 2007; Mouille *et al.*, 2007), the carbon and nitrogen nutrient balance response (Gao *et al.*, 2008), the organization pattern of root border-like cells (Durand *et al.*, 2009), and even vascular tissue formation (Fuentes *et al.*, 2010). Therefore, HG-MTs might play a crucial role in regulating the level of methylesterification and thereby the biological function of HG (Caffall and Mohnen, 2009).

Due to the observed multiple functions of AtQUA2/AtTSD2 in plant development, 28 other QUA2 family members in Arabidopsis and 25 QUA2 homologs in rice have been predicted (Mouille *et al.*, 2007), including XP-467861, a protein in rice with the highest sequence similarity to AtTSD2 (Krupková *et al.*, 2007). Rice is a model monocot and is also one of the most important crops worldwide. However, no rice HG-MTs have been reported to date. Here, we report the functional characterization of the putative methyltransferase gene *OsTSD2*, which encodes XP-467861, a member of the superfamily of class I SAM-dependent methyltransferases. Three homozygous T-DNA insertion lines of this gene were obtained for functional analyses. Botanical, biochemical, and immunochemical analyses of these T-DNA insertion lines clearly showed that *OsTSD2* functions in rice root development and cellular adhesion by affecting pectin synthesis and methylesterification.

## Materials and methods

### Plant materials and growth conditions

Three T-DNA insertion lines, *Ostsd2a* (RMD_03Z11BY36), *Ostsd2b* (RMD_05Z11BF82), and *Ostsd2c* (RMD_03Z11BN68), of rice [*Oryza sativa* ssp. *Japonica*, cultivar Zhonghua11 (ZH11)], were used for functional analysis of *OsTSD2*. Seeds were sown *in vitro* on a plate (½MS medium, 1% sucrose, 0.3% phytogel). Germinated seeds were transferred to a culture tube (½MS medium, 1% sucrose, 0.3% phytogel, with or without exogenous ABA, IAA) and grown for 4–5 d (16/8h of light/dark, 28 °C) for root phenotype analysis. For harvest of seeds, plants were grown in soil in normal growing seasons.

### Plasmid construction and plant transformation

To fuse the *Os02g51860* promoter to the *GUS* gene, the promoter of *OsTSD2*, a 1355-bp fragment upstream of the ATG of *Os02g51860* was amplified by PCR. The PCR product was cloned into pDONR207 by BP recombination (Gateway Technology; Invitrogen). After sequencing, the correct clone was introduced into the Gateway-compatible *GUS* fusion vector PHGWFS7 to produce *ProOsTSD2::GUS*. The construct was then introduced into *Agrobacterium tumefaciens* EHA105 and was transformed into the callus derived from *japonica* cultivar ZH11 by Agrobacterium-mediated transformation as previously described (Wu *et al.*, 2003).

### Real-time PCR

Gene expression analysis was performed according to method of Jin *et al.* (2015). Total RNA was extracted with an RNA extraction kit (TRIzol reagent; Invitrogen), then the first-strand cDNA was synthesized using 3 μg of RNA and 200U M-MLV reverse transcriptase (Invitrogen). Real-time PCR was performed using an optical 96-well plate in an ABI Stepone plus PCR system (Applied Biosystems) by using SYBR Premix reagent F-415 (Thermo Scientific). *OsUBI* was used as an endogenous control (Supplementary Table S1 at *JXB* online).

### GUS and Ruthenium Red staining

Histochemical analysis of the GUS reporter enzyme was performed essentially according to the method described by Jefferson *et al.* (1987). Sample tissues were incubated in reaction buffer for 2 d and the GUS staining pattern was observed under a stereomicroscope (Leica S8AP0, Germany). For acidic (unesterified) pectin detection, root tips were acquired and embedded with 5% low-melting-point agarose. Then sections (50 μm thick) were obtained with a vibratome (Leica, RM2265) and stained by 0.02% Ruthenium Red for 30min, followed by rinsing and observation under a microscope.

### Chemical analysis of pectin and ABA content

Pectin was sequentially extracted according to method of Peng *et al.* (2000). Briefly, 0.1g of dry seedling 14 d after germination was ground in phosphate-buffered saline (PBS; 0.5M, pH 7.0). Then chloroform–methanol was added to remove lipids, and DMSO was added to remove starch. When ammonium oxalate was added, pectin was obtained and the amount of uronic acids was determined by the colorimetric m-hydroxydiphenyl assay with galacturonic acid as a standard (Filisetti-Cozzi and Carpita, 1991). To determine the composition of neutral monosaccharides, pectin was hydrolyzed with 2M trifluoroacetic acid (TFA) for 1h at 121° C, with myo-inositol as the internal standard. The composition of neutral monosaccharides was determined by conversion to alditol acetates followed by GC-MS analysis (Li *et al.*, 2013).

For determination of ABA content, roots were prepared and analyzed according to method of Chen *et al.* (2013). Samples were quickly weighed, freeze-dried and crushed, followed by extraction. The lipid-soluble extracts were then absorbed and filtrated for analysis with a LC-ESI-MS/MS system (HPLC, Shim-pack UFLC SHIMADZU CBM20A system, www.shimadzu.com; MS, Applied Biosystems 4000 Q TRAP, www.appliedbiosystems.com).

### Immunofluorescence determination of degree of HG methylesterification

Roots were fixed in formaldehyde–acetic acid–ethanol (FAA, 45% ethanol, 1.85% formaldehyde, 5% glacial acetic acid) and then embedded in paraffin for sectioning. Detection of immunofluorescence was performed according to method of Dolan *et al.* (1997) with minor modifications. Briefly, sections on glass slides were blocked with 3% BSA in PBS (pH 7.2) for 30min followed by washing with PBS and incubation in monoclonal antibody supernatant LM19 (Plant Probes, 1:10 dilution, www.plantprobes.net) for 2h at 37°C. After washing with PBS, secondary anti-rat antibody conjugated to fluoresceinisothiocyanate (anti-rat/FITC, IgM, Bioss, 1:100 dilution) was applied for 1h at 37°C in the dark. Finally, sections were washed with PBS and mounted in PBS/glycerol-based anti-fade solution (5% n-propyl gallate in 90% glycerol/10% PBS) for observation using an Olympus BX61 fluorescence microscope (Olympus, Japan). As a control for background fluorescence, sections were incubated in the presence of the secondary antibody without previous incubation with the LM19 monoclonal antibody. All images were recorded with the same settings (light intensity, filters, and camera settings).

### Sectioning and microscopy

Stems were collected and freehand sectioning was carried out for anatomical observations at the cellular level. Leaves and roots were collected and fixed in FAA for 24h. After whole-staining with Ehrlich’s hematoxylin overnight, roots were rinsed with tap water for 1 d. Then serial steps of dehydration and clearing were carried out and samples were finally embedded in paraffin. Sections of 10 μm thickness were acquired on a RM 2055 rotary microtome (Leica), stained with toluidine blue, and examined using an Olympus BX61 microscope (Olympus, Japan). Toluidine blue staining was omitted for the immunofluorescence experiment.

### Root measurements

ImageJ software (https://imagej.nih.gov/ij) was used to measure the root length, diameter, lateral root density, and area of cavities. For the calculation of lateral roots, all primary roots were pre-treated with a whole-mount clearing technique (Berleth and Jürgens, 1993). Briefly, roots were mounted in a mixture of chloralhydrate/glycerol/water (8:1:3) and cleared for 2 d at room temperature. Observations were carried out under a stereo microscope (Leica S8AP0, Germany). Both emerged lateral roots and lateral root primordial were counted as lateral roots. All experiments were performed at least three times.

## Results

### Identification and characterization of a putative methyltransferase gene (*OsTSD2*) in rice

**Fig. 1.**
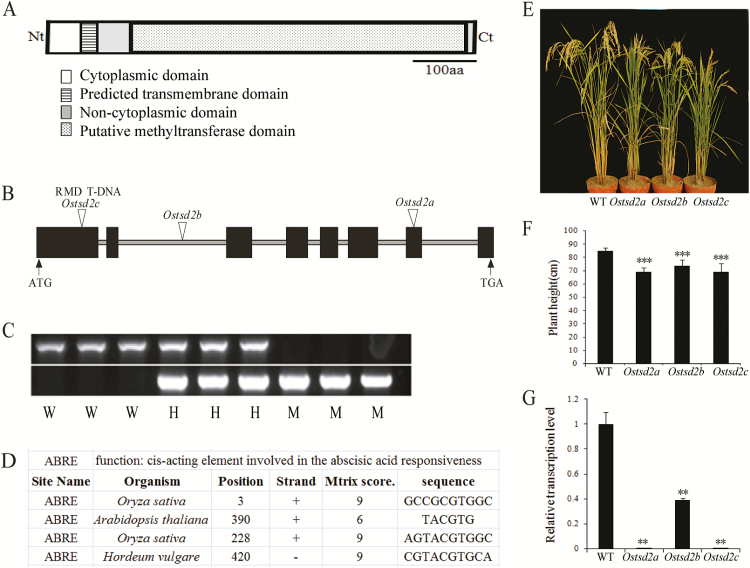
OsTSD2 protein structure, gene structure, promoter function prediction, identification, and morphological phenotype of *Ostsd2* mutants. (A) OsTSD2 protein structure. A transmembrane domain is predicted at the N-terminal (Nt). A putative methyltransferase domain is predicted at the C-terminal (Ct). (B) *OsTSD2* gene structure and T-DNA insertion lines of mutants. Exons are shown as black boxes and introns are shown as lines. The initiator ATG and stop codon TGA are shown. Triangles indicate the insertion sites of three T-DNA lines. (C) Identification of *Ostsd2* mutants by PCR. W, wildtype; H, heterozygous; M, homozygous. (D) Four ABREs were found in *OsTSD2* using the promoter functional prediction tool (PlantCARE). (E) Morphological phenotype of three *Ostsd2* mutants compared with the wildtype (WT, ZH11) at the mature stage. (F) Plant height of WT and three *Ostsd2* mutants. (G) *OsTSD2* transcription levels in roots of WT and three mutants. Results of Student’s *t*-test: *, *P*<0.05; **, *P*<0.01; ***, *P*<0.001. (This figure is available in color at *JXB* online.)

Because of the important role of HG-MTs and the pleiotropism of *AtTSD2* in plant development (Krupková *et al.*, 2007; Durand *et al.*, 2009; Fuentes *et al.*, 2010), we aimed to identify a putative HG-MT gene in rice. Comparison of the protein sequence of AtTSD2 (AT1G78240) with the rice genome sequence revealed that it was most similar (58%) to the putative protein Os02g51860 (XP-467861; Krupková *et al.*, 2007) (Supplementary Fig. S1 and Fig. 1A), and the corresponding locus was named *OsTSD2* in this study. The *OsTSD2* genomic DNA consists of eight exons and seven introns (Fig. 1B), encoding a polypeptide of 660 amino acids (Fig. 1A) with a predicted molecular weight of 73.61kDa and a pI of 6.51. The transmembrane hidden Markov model (TMHMM) prediction server (http://www.cbs.dtu.dk/services/TMHMM-2.0/) predicted that OsTSD2 is a type II transmembrane protein that consists of a short cytoplasmic *N*-terminus followed by a single transmembrane helix and a long non-cytoplasmic *C*-terminus. A search for structural domains revealed the presence of an SAM-dependent methyltransferase domain (Methyltransf-29) (Fig. 1A) and classified OsTSD2 as a member of the superfamily of SAM-dependent methyltransferases, class I (http://www.ncbi.nlm.nih.Gov/Structure/cdd/wrpsb.cgi). OsTSD2 is predicted to be localized in the Golgi body (http://www.softberry.com/berry.phtml?topic=protcompan&group=programs&subgroup=proloc; Integral Prediction of protein location: Membrane-bound Golgi with score 7.4), which is consistent with its putative role at the site of cell wall polysaccharide synthesis (Ridley *et al.*, 2001). In addition, prediction of the function of its promoter indicated that there are four abscisic acid response elements (ABREs) in the 1500bp before the translational start codon (http://bioinformatics.psb.ugent.be/webtools/plantcare/html/) (Fig. 1D), thus suggesting that *OsTSD2* might be involved in the ABA response (Fujita *et al.*, 2013).

### Mutation of *OsTSD2* induced a dwarf phenotype and deficiency in cellular adhesion in the root

To determine the function of *OsTSD2* in plant development, three independent T-DNA insertion lines (Os*tsd2a*, Os*tsd2b*, and Os*tsd2c*) were acquired from the Rice Mutant Database (Zhang *et al.*, 2006), according to flanking sequences provided by RiceGE (http://signal.salk.edu/cgi-bin/RiceGE). PCR analysis revealed that each of the three lines contained an insertion within the *OsTSD2* gene. Further sequence analyses showed that T-DNAs were inserted in the 7th exon, 2nd intron, and 1st exon of the *OsTSD2* gene in Os*tsd2a*, Os*tsd2b*, and Os*tsd2c*, respectively (Fig. 1B). Morphologically, all of the mutants displayed a dwarf phenotype (Fig. 1E, F). All of the heterozygous insertion lines showed segregation of seedlings exhibiting the dwarf phenotype. The T_1_ family of the *Ostsd2a* mutant line produced 53 wildtype-like plants and 19 mutant plants (with an expected ratio of 54:18 for 3:1 segregation, *χ* ^2^=0.0364, *P*=0.849). All of the seedlings that exhibited the mutant phenotype were found to be homozygous for the T-DNA insertion in *OsTSD2* via PCR (Fig. 1C), thus confirming that the mutation segregates as a recessive allele. Because Os*tsd2a*, Os*tsd2b*, and Os*tsd2c* caused similar phenotypic changes, the mutant phenotypes described here were believed to be due to null or knockdown mutations, as indicated by abolished or decreased transcription of *OsTSD2* in the three mutant lines (Fig. 1G).

**Fig. 2.**
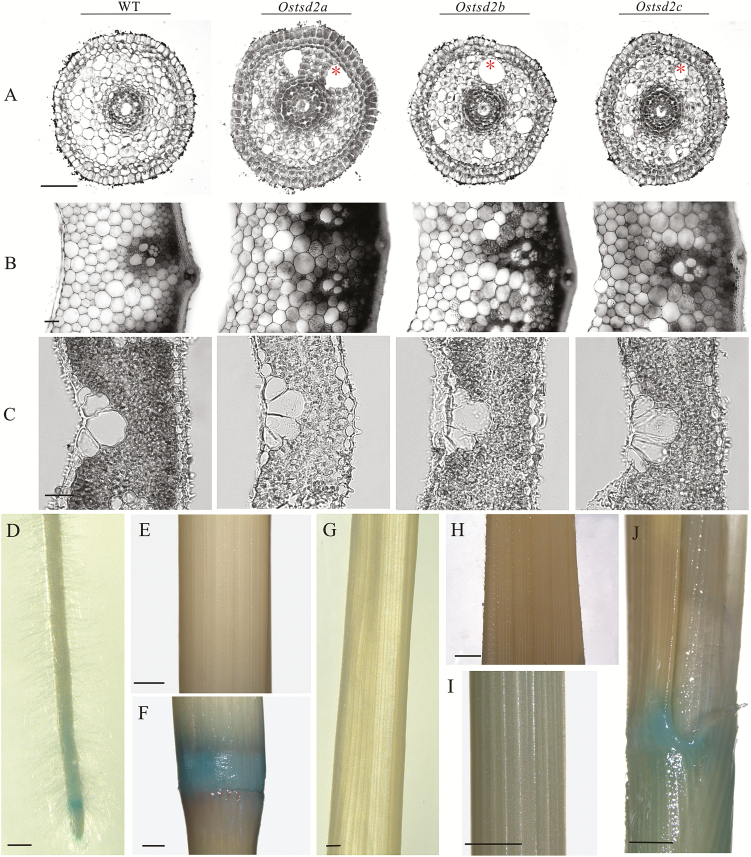
Cellular morphology of vegetative organs of *Ostsd2* mutants and expression pattern of *OsTSD2*. (A–C) Transverse sections of three vegetative organs. Scale bars are 50 μm. (A)The elongation zone of the root, (B) stem internode, and (C) young leaf blade (7 d after germination, DAG). (D–J) GUS staining in different vegetative organs of the *ProOsTSD2::GUS* transformed line. Scale bars are 0.5mm. (D) The primary root tip of a seedling at 7 DAG. (E) Internode of the stem at the flowering stage. (F) Node of the stem at the flowering stage. (G) Leaf blade of a seedling at 7 DAG. (H) Leaf blade of the flag leaf at the flowering stage. (I) Sheath of the flag leaf at the flowering stage. (J) Joint of the flag leaf at the flowering stage. (This figure is available in color at *JXB* online.)

Because pectin methyltransferases have been functionally related to cellular adhesion (Frank *et al.*, 2002; Krupková *et al.*, 2007), we wondered whether mutation of *OsTSD2* might cause a similar deficiency in rice. To this end, transverse sectioning of different vegetative organs, including roots, stems, and leaves, was performed in order to detect cellular changes. In the Os*tsd2* mutants, alteration of cellular adhesion occurred in the root (Fig. 2A) but not in stem internodes (Fig. 2B) and leaf blades (Fig. 2C). A deficiency in the cellular adhesion of Os*tsd2* mutants was recognized by the observation of large cavities in the pre-cortex in the elongation zone of the root, whereas these cells were tightly connected in the wildtype (ZH11) plants (Fig. 2A). Because the changes were specific to the roots, it suggested that *OsTSD2* may function differently in different tissues/organs. The *proOsTSD2::GUS* vector was then constructed, and positive transgenic lines were acquired to explore the expression pattern of *OsTSD2* in plants. GUS staining revealed that *OsTSD2* transcription showed specificity between different tissues/organs. Of the three organs examined, transcription occurred in the root tip (Fig. 2D), but not in stem internodes (Fig. 2E) and leaf blades/sheaths (Fig. 2G–I), although transcription of *OsTSD2* occurred in stem nodes (Fig. 2F) and leaf joints (Fig. 2J). This transcription pattern was functionally consistent with a deficiency in cellular adhesion in the roots of the *Ostsd2* mutants.

### Mutation of *OsTSD2* altered root development and induced more swollen root tips in response to exogenous ABA

**Fig. 3.**
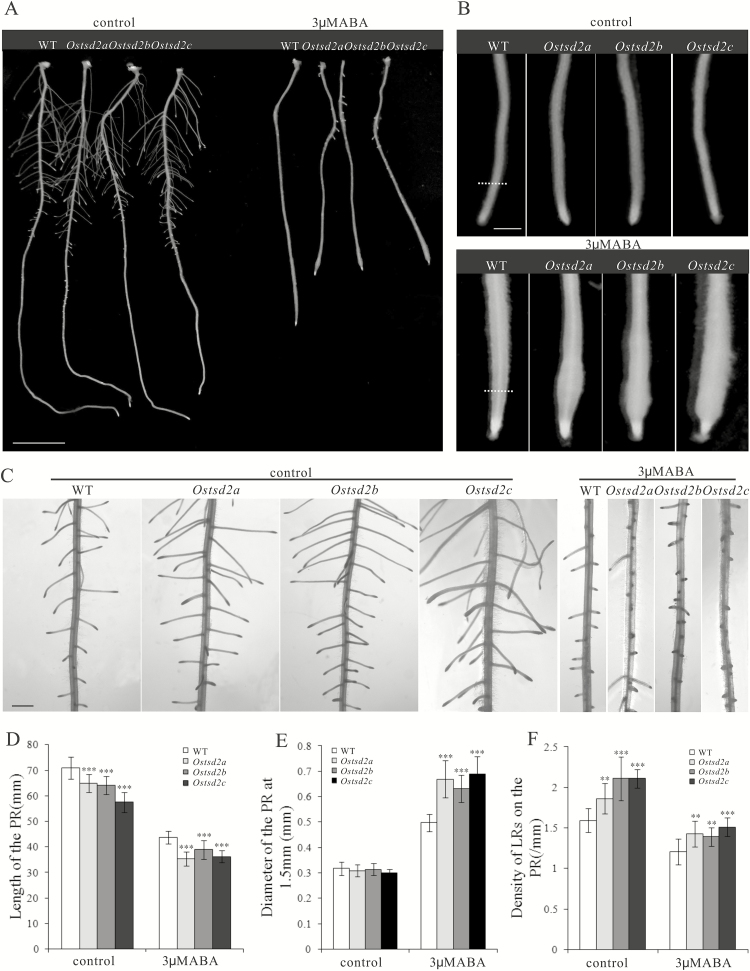
Root development and response to exogenous ABA of *Ostsd2* mutants. (A) Morphology of the primary root (PR) of WT and three *Ostsd2* mutants at 8 d after germination (DAG). Scale bar = 1cm. (B) Morphology of the root tips of the PR. Scale bar = 1mm. (C) Distribution pattern of lateral roots (LRs) on the PR. Scale bar = 1mm. (D) Mean length of the PR. (E) Mean diameter of the PR at 1.5mm from the root tip (dashed line in B). (F) Mean densitity of LRs on the PR. Results of Student’s *t*-test: **, *P*<0.01; ***, *P*<0.001.

To determine whether *OsTSD2* is involved in the ABA response, we germinated seeds of *Ostsd2* mutants and transferred them to MS medium either with or without exogenous ABA, with wildtype (WT) seeds grown on the same medium as a control. Seedlings of the *Ostsd2* mutants showed short primary roots (Fig. 3A, D) and more lateral roots compared with WT (Fig. 3C, F) in the absence of exogenous ABA. Upon treatment with exogenous ABA, *Ostsd2* mutants showed inhibited elongation of the primary root (Fig. 3A, D) and reduced lateral root density (Fig. 3C, F); both of these responses were just the same as in the WT and are seen as typical responses to ABA (De Smet *et al.*, 2006; Tseng *et al.*, 2013), thus implying that the capacity to respond to ABA was not damaged in the *Ostsd2* mutants. The *Ostsd2* mutants while exhibited root tips that were more swollen than in the WT after application of exogenous ABA (Fig. 3B, E), which is a novel function of ABA that has previously been reported in Taichung native 1 rice (Chen *et al.*, 2006), suggesting that the roots of the *Ostsd2* mutants have an exaggerated response to ABA in transverse growth of the root.

### Mutation of *OsTSD2* affects the pattern of aerenchyma formation and ABA can partially restore cellular adhesion in roots of *Ostsd2* mutants

To more clearly understand the relationship between the deficiency in cellular adhesion and alterations in root development in *Ostsd2* mutants, we made both longitudinal and transverse sections of *Ostsd2* roots with or without ABA treatment to observe the cellular changes. Data from the longitudinal sections showed that the form and size of cells in the elongation zone were similar between *Ostsd2* and WT plants in the absence of exogenous ABA (see Supplementary Fig. S2, control). After treatment with 3 μM ABA, the meristematic zone was significantly shortened, and cells in the elongation zone appeared enlarged, which substantially contributed to the swollen forms of both WT and *Ostsd2a* roots (see Supplementary Fig. S2, 3 μM ABA). Furthermore, in *Ostsd2* the cells in the elongation zone were more squashed; in other words, the value of the ratio of the longitudinal/transverse axis width was lower than in WT plants (Supplementary Fig. S3). Therefore, the difference in the degree of swelling may be partially due to excessive growth in the transverse direction.

**Fig. 4.**
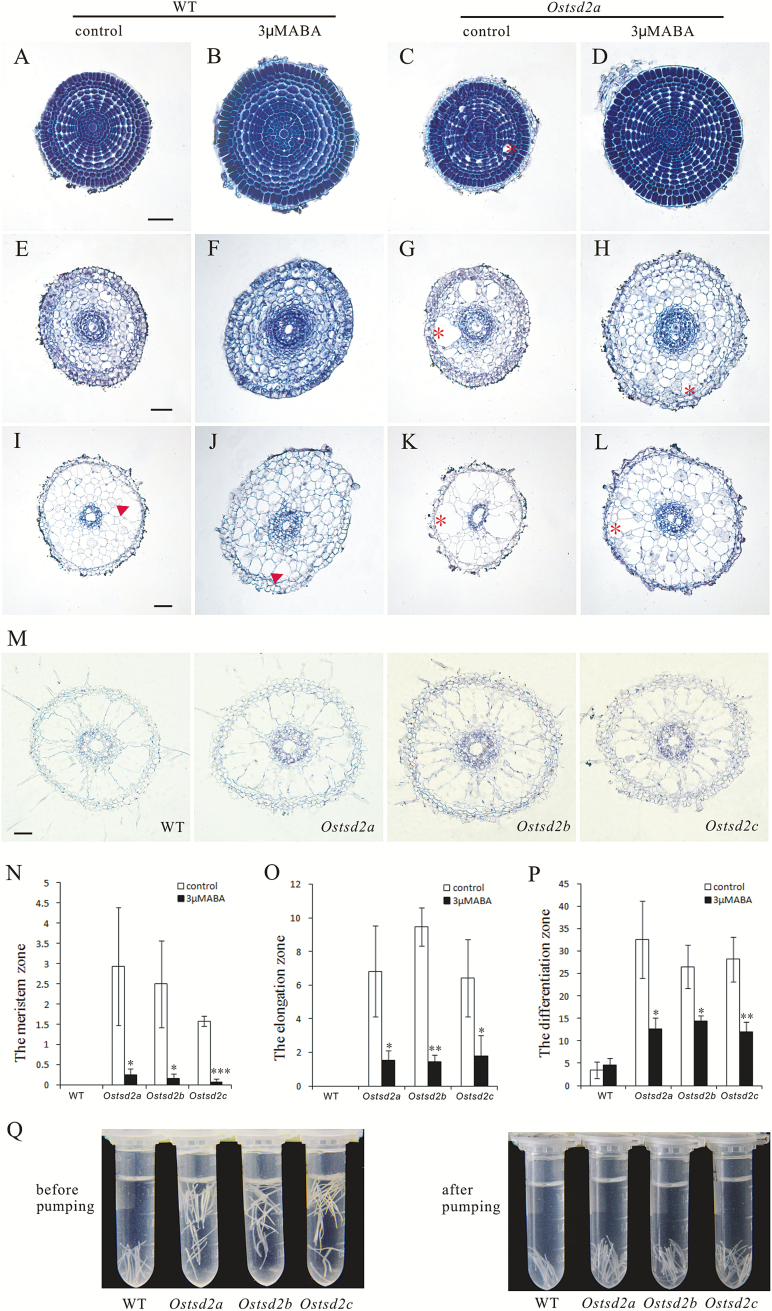
Changes in aerenchyma formation and root tip density of *Ostsd2* mutants. (A–M) Sequential transverse sectioning of roots in the wildtype (WT) and *Ostsd2a* mutants without (control) or with exogenous 3 μM ABA treatment. All samples were whole-stained with Ehrlich’s hematoxylin before embedding and transverse sectioning at a thickness of 10 μm. Arrowheads indicate lysigenous aerenchyma (I, J). Asterisks indicate schizogenous aerenchyma (C, G, H, K, L). Scale bars are 50 μm (shown in A for A–D; shown in E for E–H; shown in I for I–L). (A–D) Sections of the meristematic zone. (E–H) Sections of the elongation zone. (I–L) Sections of the differentiation zone. (M) Sections of the completely mature zone about 2cm from the root cap. (N) Proportion of cavities in the transverse area in the meristematic zone (as in A–D). (O) Proportion of cavities in the transverse area in the elongation zone (as in E–H). (P) Proportion of cavities in the transverse area in the differentiation zone (as in I–L). Results of Student’s *t*-test: *, *P*<0.1; **, *P*<0.01; ***, *P*<0.001. (Q) Difference in root tip density between WT plants and *Ostsd2* mutants in a fixative solution of FAA before and after pumping for 20min. (This figure is available in color at *JXB* online.)

We also examined the transverse sections because the number of cell layers might also contribute to the difference in root diameter between *Ostsd2* mutants and WT plants. Because the cellular organization in the end of the meristematic zone is very regular and the number of cell layers is maintained in the following cell elongation and differentiation process, we examined and calculated the cell layers in this zone and found that there was no difference between the *Ostsd2* mutants and WT plants (both included 8–9 cell layers in the ground meristem with or without ABA treatment, *n*=10). Consistent with the cellular adhesion changes shown in Fig. 2A, ‘schizogenous aerenchyma’ appeared in the roots of *Ostsd2* mutants even from the end of the meristematic zone (Fig. 4C, asterisk; see also Supplementary Figs S2, S4) but not in WT (Fig. 4A). It has been reported that rice roots form only lysigenous aerenchyma upon the death and subsequent lysis of some cells inside the cortex of the mature zone (Jackson *et al.*, 1985). However, in the meristematic zone of *Ostsd2*, the formation of cavities began with significantly expanded intercellular spaces between lines of pre-cortical cells (Fig. 4C; Supplementary Fig. S4); therefore, we called these cavities ‘schizogenous aerenchyma’ rather than ‘lysigenous aerenchyma’ (Evans, 2003). With cell elongation, the cavities of ‘schizogenous aerenchyma’ became bigger and more striking (Fig. 4G; Supplementary Fig. S4). Statistical analysis of the proportion of cavity area in transverse sections showed a significant difference between the three *Ostsd2* lines and the WT in both the meristematic and elongation zone (Fig. 4A , C, E, G, N, O). Coupled with the later appearance of lysigenous aerenchyma, the proportion of aerenchyma area in transverse sections was still significantly higher in the differentiation zone of the *Ostsd2* mutants (Fig. 4K, P), whereas only lysigenous aerenchyma formed sporadically in this zone of WT plants (Fig. 4I, P). Moreover, in the absence of exogenous ABA in the *Ostsd2* mutants (Fig. 4C, G, K), the schizogenous aerenchyma was distributed in an irregular manner, and the sizes of the cavities differed significantly. However, after treatment with ABA (Fig. 4D, H, L), the schizogenous aerenchyma became less distinct because the cavities became smaller, and the sizes of the cavities were roughly consistent; in other words, the appearance of schizogenous aerenchyma in the *Ostsd2* mutants was partially restored by ABA treatment (Fig. 4N–P). This type of difference was not found in WT plants regardless of whether or not they were treated with exogenous ABA (compare Fig. 4A, E, I with B, F, J). Because schizogenous aerenchyma develops from cell separation and differential cell expansion that creates spaces between cells (Evans, 2003), the formation of this type of aerenchyma in the roots of *Ostsd2* mutants showed that the *OsTSD2* gene is tightly linked to cellular adhesion, a process that may be partially linked to ABA. Interestingly, the final pattern of aerenchyma in the completely mature zone (about 2cm from the tip) of the *Ostsd2* plants was similar to that of the WT (Fig. 4M), implying that mutation of *OsTSD2* did not disturb the formation of lysigenous aerenchyma, since the quantity of schizogenous aerenchyma is relatively less than that of lysigenous aerenchyma, and the schizogenous aerenchyma would not form plenty of cavities in that way.

When root tips (about 1cm in length) were placed in FAA solution for fixation, WT roots sank whilst *Ostsd2* roots were suspended (Fig. 4Q, before pumping), thus suggesting that a difference in their root densities might exist. To further investigate this, air was pumped out of the solution, and the roots of all lines sank to the bottom of the tubes (Fig. 4Q, after pumping); this observation indicated that the differences in root density resulted from the existence of a large amount of air located in the schizogenous aerenchyma in the roots of the *Ostsd2* mutants.

### Mutation of *OsTSD2* affects root development by reducing the degree of HG methylesterification

**Fig. 5.**
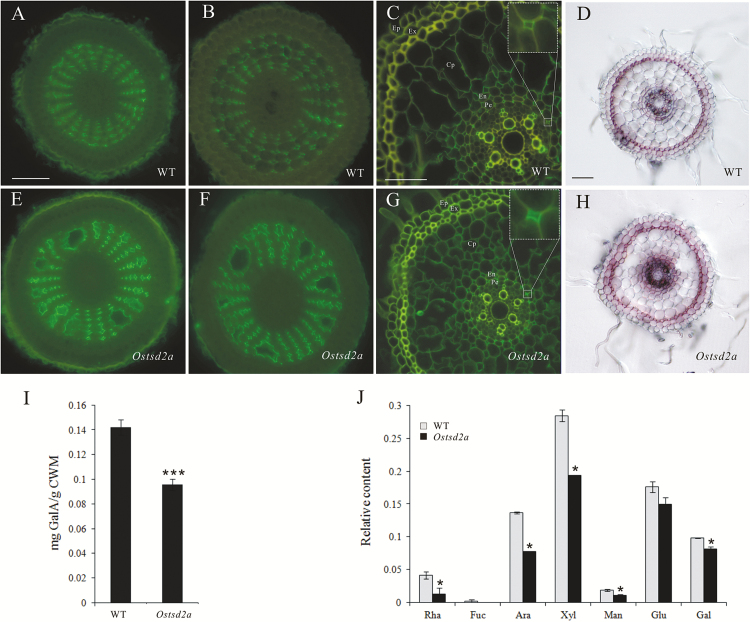
Changes in HG methylesterification patterns and content of pectin in *Ostsd2a* mutants. (A–C, E–G) Immunofluorescent detection of HG methylesterification patterns using the monoclonal antibody LM19. All samples were embedded and sectioned transversely at 10 μm thickness. All sections were incubated with the monoclonal antibody LM19 and then the secondary antibody conjugated to FITC. The control (with LM19 omitted) is shown in Supplementary Fig. S5. Scale bars are 50 μm (shown in A for A, B, E, F; shown in D for D, H) and 25 μm (shown in C for C, G). (A, E) Sections of the meristematic zone, (B, F) sections of the elongation zone, and (C, G) sections of the maturation zone. The insets in (C, G) show close-ups of the intercellular corners between pericycle cells (Pe) and the neighboring endoderm cells (En). Other abbreviations: Ep, epidermis; Ex, exodermis; Cp, cortex parenchyma. (D, H) Ruthenium Red staining. Transverse sections of the differentiation zone were acquired by use of a vibratome and stained with 0.02% Ruthenium Red. Obvious staining mainly occurs in the exodermis of both lines. Scale bar = 50 μm. (I) Content of GalA in the pectin of the cell walls of the seedling. (J) Relative content of monosaccharide composition in pectin extracted from seedlings according to the peak area detected by GC-MS. Abbreviations: Rha, rhamnose; Fuc, fucose; Ara, arabinose; Xyl, xylose; Man, mannose; Glu, glucose; Gal, galactose. Reuslts of Student’s *t*-test: *, *P*<0.1; **, *P*<0.001. (This figure is available in color at *JXB* online.)

Because pectins play important roles in cellular adhesion/cell division, and *OsTSD2* encodes a putative methyltransferase, we tested whether the level of pectin methylesterification was affected in the roots of *Ostsd2* mutants by immunofluorescence using the monoclonal antibody LM19, which binds strongly to unesterified HG domains of pectic polysaccharides (Verhertbruggen *et al.*, 2009). Phenotypes of the roots of *Ostsd2* mutants included the formation of schizogenous aerenchyma in the meristematic zones, more swollen cells in the elongation zone after treatment with exogenous ABA, and more lateral root formation in the mature zone; therefore, sections of these three zones in the roots of WT and *Ostsd2a* plants were all examined using LM19. Unesterified HG is normally mainly distributed in the intercellular spaces of the cortical parenchyma in rice roots (Knox *et al.*, 1990; Yang *et al.*, 2008), implying that the unesterified HG epitope has an important role in the regulation of the function of these cells (Javis *et al.*, 2003). Overall, signal intensities as a result of LM19 labeling were stronger in *Ostsd2a* plants (Fig. 5E–G) than in WT plants (Fig. 5A–C) in all three zones.

In both the meristematic (Fig. 5A, E) and elongation zones (Fig. 5B, F), the radial and regular distributions of lines of cortical cells were paralleled by the orderly distribution of LM19 labeling in intercellular spaces among the cortical parenchyma cells in WT plants (Fig. 5A, B). However, in the *Ostsd2a* line, stronger signal intensity by LM19 labeling was observed, and the regular pattern of arrangement was changed because of the presence of schizogenous aerenchyma (Fig. 5E, F). Strong LM19 labeling was observed alongside the cavity created by the schizogenous aerenchyma, which was not observed in WT plants (Fig. 5A, B). Similar alterations were also found in the roots of *Ostsd2b* plants (Supplementary Fig. S5). These findings suggest that the formation of schizogenous aerenchyma in *Ostsd2* roots is coupled with increased unesterified HG and a deficiency in cellular adhesion. In the mature zone (Fig. 5C, G), the stronger signal intensity by LM19 labeling was detected not only in the intercellular spaces of the cortical parenchyma but also in the intercellular spaces between pericycle cells (magnified in the insets in Fig. 5), which were related to lateral root initiation by cell division coupled with cell wall reconstruction (De Smet *et al.*, 2006).

Ruthenium Red, a dye that detects acidic (unesterified) pectin (Hanke and Northcote, 1975), was also used to detect differences between WT plants and *Ostsd2* mutants. The results in the mature zone (1mm from the root tip) showed that staining in *Ostsd2a* roots (Fig. 5H) was increased compared with WT roots (Fig. 5D), although the staining mainly occurred in the exodermis and not in the cortical parenchyma (Laskowski *et al.*, 2006).

### Mutation of *OsTSD2* affects the HG content

Because deficiencies in cellular adhesion have been frequently reported in conjunction with deficiencies in pectin synthesis (Bouton *et al.*, 2002; Mouille *et al.*, 2007), we tested whether the cell walls of *Ostsd2* seedlings showed alterations in pectin content and monosaccharide composition. After a series of fractionation steps, pectin was obtained, and the content of its main component, GalA, was measured in a colorimetric m-hydroxydiphenyl assay (Filisetti-Cozzi and Carpita, 1991). GalA was significantly less abundant in *Ostsd2a* mutants compared with WT plants (Fig. 5I). To quantify the monosaccharide composition, we performed a subsequent acid hydrolysis of pectin with trifluoroacetic acid and derivatization of neutral monosaccharides to alditol acetates followed by gas chromatography/mass spectrometry (GC-MS) analysis. The results indicated that the content of neutral monosaccharides in pectin was also decreased in *Ostsd2a* plants (Fig. 5J).

### Relationship between *OsTSD2* transcription and ABA in roots

**Fig. 6.**
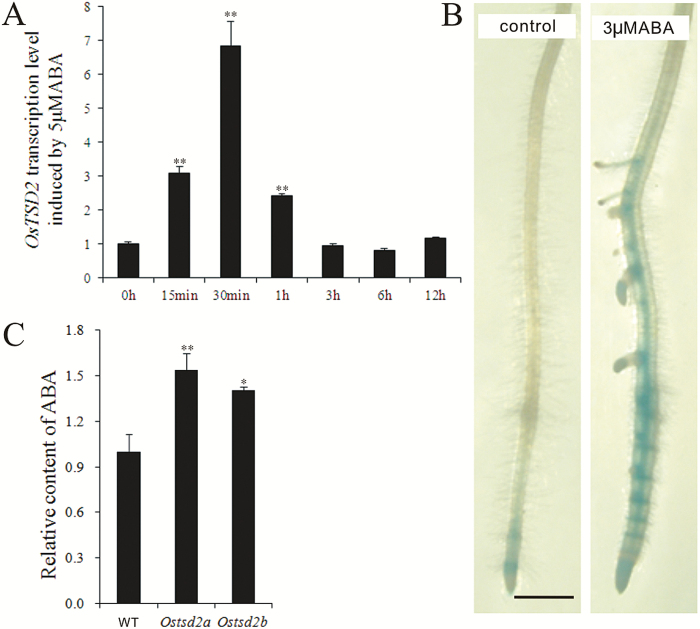
ABA inducement on *OsTSD2* transcription and relative content of endogenous ABA. (A) Relative transcription level of *OsTSD2* induced by exogenous 5μM ABA over time (min, minute; h, hour). The values were standardized according to the endogenous control gene *OsUB*I and the value at 0h is expressed as 1. (B) Expression of *OsTSD2* in the primary root tip of the pro*OsTSD2:GUS* transformed line without (left, control) and with (right) previous treatment with exogenous 3μM ABA. Scale bar = 1mm. (C) Relative content of endogenous ABA in roots of WT, *Ostsd2a*, and *Ostsd2b* plants according to the peak area detected by LC-ESI-MS/MS. Results of Student’s *t*-test: *, *P*<0.05; **, *P*<0.01. (This figure is available in color at *JXB* online.)

The normal responses of *Ostsd2* mutants to the application of exogenous ABA, including reductions in root length and lateral root density, and the exaggerated formation of swollen root tips, make it difficult to determine whether *OsTSD2* is involved in ABA synthesis/signaling. A similar confusing situation also occurs in the *epc1* mutant, in which root elongation exhibits an exaggerated response to exogenous ABA, whereas the application of exogenous ABA has no significant effect on the level of *EPC1* gene expression (Bown *et al.*, 2007). In the *Attsd2* mutant, genetic interaction analyses involving ABA INSENSITIVE4, an APETALA2-type transcription factor required for ABA inhibition of germination, did not support a role for *AtTSD2* in the ABA signaling pathway (Finkelstein, 1994; Finkelstein *et al.*, 1998; Fuentes *et al.*, 2010). Therefore, we attempted to explore the relationship between *OsTSD2* transcription and the ABA synthesis /signaling pathways. Treatment with exogenous 5 μM ABA and real-time PCR analysis revealed that *OsTSD2* was transcriptionally responsive to ABA in roots within 15min (Fig. 6A), consistent with the results of the promoter analysis by PlantCARE (Fig. 1D), and was further confirmed by the enhanced expression in roots in the *proOsTSD2::GUS* line after treatment with exogenous ABA (Fig. 6B). After methanol extraction and detection using high-performance liquid chromatography (HPLC), we determined the relative content of ABA and found that it was increased in the roots of the *Ostsd2* mutants (Fig. 6C), which was consistent with increased transcription levels of most genes involved in ABA synthesis (Supplementary Fig. S6A; Ye *et al.*, 2012). In addition, changes in the transcriptional levels of the transcription factor genes involved in the ABA signaling pathway were disordered in *Ostsd2* mutants (Supplementary Fig. S6B; Xiang *et al.*, 2008; Lu *et al.*, 2009; Sperotto *et al.*, 2009). These findings suggest that there may be a feedback inhibition effect of *OsTSD2* transcription on ABA synthesis. The more swollen root tips of the *Ostsd2* mutants upon treatment with exogenous ABA may be partially caused by increased ABA content; however, its dependence on changes in ABA signaling remain uncertain.

### Exogenous IAA can accelerate the deficiency in cellular adhesion of *Ostsd2* mutant roots

**Fig. 7.**
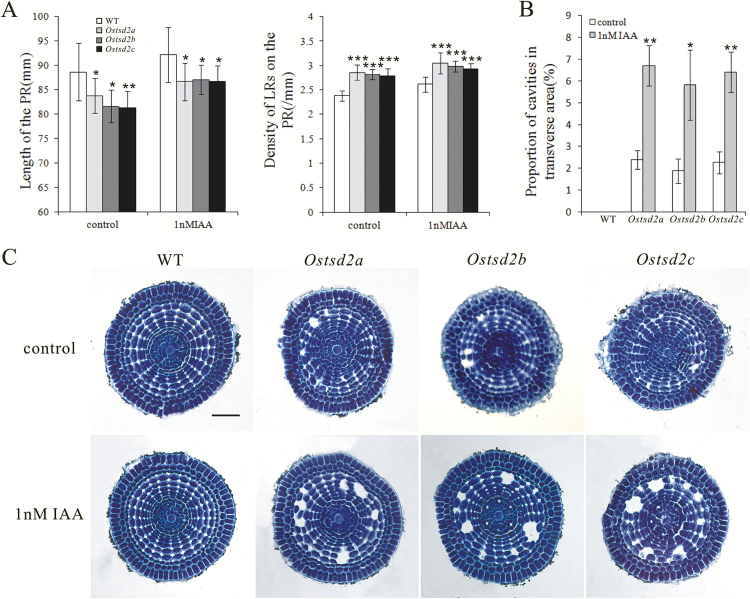
Effect of exogenous IAA on root development and formation of schizogenous aerenchyma in the meristematic zone of the root in three *Ostsd2* mutant lines. 1n M IAA was used to treat the roots for 4 d and then root length and lateral root density were measured. Root tips were collected for embedding and sectioning. (A) Mean length of the primary root (PR) and lateral root (LR) densities on the PR. (B) Mean proportion of cavities in the transverse area in the primary meristematic zone of WT and three *Ostsd2* mutants. Results of Student’s *t*-test: *, *P*<0.1; **, *P*<0.01; ***, *P*<0.001. (C) Images of transverse sections of the primary meristem zone of WT and three *Ostsd2* mutants. Scale bar = 50 μm. (This figure is available in color at *JXB* online.)

Auxin, the universal hormone of plants, usually plays opposite roles to ABA and is generally a positive factor for root growth, including root elongation and lateral root formation (De Smet *et al.*, 2006; Ding *et al.*, 2015; Subramaniam *et al.*, 2016). ABA has been found to regulate root growth under osmotic stress conditions via an interacting hormonal network with cytokinin, ethylene, and auxin (Rowe *et al.*, 2016). So we wondered whether the roots of *Ostsd2* mutants could respond normally to exogenous IAA. Interestingly, both improved root elongation and lateral root formation were seen in *Ostsd2* mutants upon treatment with exogenous IAA, just the same as in the WT (Fig. 7A). This suggested that the capacity to respond to IAA was also generally not damaged in the *Ostsd2* mutants. In addition, cellular observation of the end of the primary meristematic zone showed that IAA can accelerate the deficiency in cellular adhesion and the formation of more striking schizogenous aerenchyma in the roots of the *Ostsd2* mutants (Fig. 7B, C). The opposite effects of ABA and IAA on cellular adhesion of the *Ostsd2* roots confirm the deficiency of cellular adhesion due to mutation of *OsTSD2* and its involvement in pectin modification.

## Discussion

### *OsTSD2* functions as a putative HG-MT gene

As the enzyme that catalyzes the addition of methyl groups to HG, HG-MT is crucial for determining the extent and pattern of HG methylesterification and, ultimately, the biological functions of pectin (Caffall and Mohnen, 2009). The catalytic activity or function of HG-MTs has been detected and partially characterized in several dicot species (Kauss and Hassid, 1967; Vannier *et al.*, 1992; Goubet *et al.*, 1998; Ishikawa *et al.*, 2000; Ibar and Orellana, 2007; Krupková *et al.*, 2007). However, to date, no HG-MT has been reported in monocots. Here, we report that OsTSD2 functions as a putative HG-MT with an important role in regulating plant development in monocots, even though they contain considerably less pectin (Caffall and Mohnen, 2009). Analysis suggested that OsTSD2 is a Golgi-localized type II membrane protein that belongs to the superfamily of *S*-adenosyl-L-methionine-dependent methyltransferases with a plant-specific, putative methyltransferase domain. Mutation of *OsTSD2* results in dwarf plants and abnormal cellular adhesion; both of these phenotypes are typical of mutants that are deficient in pectin (Bouton *et al.*, 2002; Singh *et al.*, 2005; Mouille *et al.*, 2007). Biochemically, there was increased unesterified HG and reduced GalA and neutral monosaccharides in the pectin of Os*tsd2a* plants. Notably, mutation of *OsTSD2* resulted in the alteration of root development, including shorter root elongation, the appearance of more lateral roots, and an altered pattern of aerenchyma formation. These results strongly support the notion that OsTSD2 functions as a HG-MT that plays an important role in rice root development and may be functionally different from its homolog, AtTSD2. The function of OsTSD2 elucidated here is consistent with the potential role of HG-MTs in affecting the methylesterification status of HG, thereby influencing plant development (Roberts, 1990; Guglielmino *et al.*, 1997; Caffall and Mohnen, 2009).

### *OsTSD2* is required for root development in rice

Several pathways are involved in rice root development (Xia *et al.*, 2014). Here, we report on *OsTSD2* as a putative HG-MT gene required for root development. Due to the increased unesterified HG and decreased pectin contents, cellular adhesion was affected, and shorter primary roots and increased numbers of lateral roots were observed in *Ostsd2* mutants. In Arabidopsis, mutation of *AtTSD2* has been reported to impair both primary root growth and lateral root formation (Krupková *et al.*, 2007). Mutation of *AtEPC1* (*ECTOPICALLY PARTING CELLS 1*), a glycosyltransferase (GT64) gene, has been reported to reduce primary root elongation but to increase lateral root density in 10-d-old seedlings (Singh *et al.*, 2005). In studies of root responses to environmental stresses, such as H_2_O_2_ (Xiong *et al.*, 2015), ammonium (Wang *et al.*, 2015), and cadmium (Xiong *et al.*, 2009), inhibited root elongation is usually coupled with increased pectin content and increased demethylesterification levels. Although there are still conflicting results regarding the exact role of pectin in root development, studies have shown that root development is dependent on cell extension/division, which is tightly linked to cellular adhesion and pectin activities inside the cell wall. We have demonstrated the involvement of both ABA and IAA in controlling root development when *OsTSD2* was mutated. The normal responses in root elongation and lateral root formation indicated that mutation of *OsTSD2* generally does not affect the root’s response capacity to both ABA and IAA. And for the meristematic and elongation zones, where deficiency in cellular adhesion occurs due to mutations of *OsTSD2*, the restoration effect of ABA and the exacerbation effect of IAA confirm the control by *OsTSD2* on pectin and cellular adhesion, which are both closely related to cell growth.

Aerenchyma formation is a morphological change that occurs constitutively in plants or when they are subject to flooded or hypoxic conditions. It is known to enhance the internal diffusion of atmospheric and photosynthetic oxygen from the aerial parts to the roots, allowing them to maintain aerobic respiration (Armstrong, 1980). In general, aerenchyma can be classified into two types: (1) schizogenous aerenchyma, which develops by cell separation and differential cell expansion that creates spaces between cells, and (2) lysigenous aerenchyma, which results from the death and subsequent lysis of certain cells (Evans, 2003). In rice, a typical wetland plant, lysigenous aerenchyma can constitutively form in roots, and cell death begins in the cells of the mid-cortex region and then spreads out radially to the surrounding cortical cells (Kawai *et al.*, 1998); the epidermis, hypodermis/exodermis, endodermis, and stele are unaffected, indicating that lysigenous aerenchyma formation occurs via tightly controlled mechanisms (Yamauchi *et al.*, 2013). In the final stage of lysigenous aerenchyma formation, cell wall degradation occurs because of the combined actions of pectolytic, xylanolytic, and cellulolytic enzymes (Jackson and Armstrong, 1999; Evans, 2003). Changes in distribution of esterified and de-esterified pectins in the walls of cells in the maize cortex have been observed during cell death and aerenchyma formation initiated by hypoxia (Gunawardena *et al.*, 2001), thus suggesting that pectin is involved in lysigenous aerenchyma formation. Here, we report the formation of schizogenous aerenchyma in the root cortex of rice due to mutation of *OsTSD2* and a deficiency in cellular adhesion. Interestingly, the formation of schizogenous aerenchyma in the *Ostsd2* mutant also begins in the mid-cortex, which is consistent with the origin of future lysigenous aerenchyma (Kawai *et al.*, 1998). Therefore, it is possible that the formation of both types of aerenchyma may share similar mechanisms in the *Ostsd2* mutants. Compared with the detailed studies of the formation of lysigenous aerenchyma (Voesenek *et al.*, 2006), information on the regulation of schizogenous aerenchyma formation remains scant. Our present results may be helpful in understanding the formation of schizogenous aerenchyma.

### The degree of unmethylesterified pectin plays multiple roles in root development in a zone-dependent manner

It has been suggested that the functional characteristics of pectin are affected by the extent and pattern of its methylesterification (Jarvis *et al.*, 2003; Wolf *et al.*, 2012). A minimum stretch of nine unmethylesterified galacturonic acid residues can form Ca^2+^ linkages, which may promote the formation of an ‘egg-box’ model structure (Liners *et al.*, 1992). The presence of these ‘egg-box’ structures is assumed to induce gel formation and thus strengthen the cell wall or become a target for pectin-degrading enzymes, such as polygalacturonases and pectin/pectate lyases (Wolf *et al.*, 2009). Multiple lines of evidence have verified that the degree of methylesterification can vary from one cell type to another or in a tissue-specific manner (Knox *et al.*, 1990; Dolan *et al.*, 1997; Sobry *et al.*, 2005; Wolf *et al.*, 2012). Thus, the degree of pectin methylesterification should be under spatial regulation in developing tissues and should be matched synergistically with cell development in specific tissues. The plant root, a typical model for elucidating development mechanisms, is composed of four functional zones: the root cap, the meristematic zone, the elongation zone, and the mature zone. Because each zone consists of at least three distinct layers, the root is an ideal system to reveal the role of pectin modification. Given the sequential changes in the different zones of the root in response to mutation of *OsTSD2* and the alterations in pectin content and level of methylesterification, along with the opposite roles of ABA and IAA in root development, we propose a hypothesis to describe the relationship among *OsTSD2*, HG methylesterification, and root development based on pectin’s control of cell growth/division.

In performing its function related to cellular adhesion, the unmethylesterified pectin is usually located in the cell wall. Regardless of the origin of the unmethylesterified pectin, whether transported in a highly methylesterified form and then demethylesterified by PME in the cell wall or directly transported from the Golgi bodies in a less methylesterified form (Guglielmino *et al.*, 1997), the unmethylesterified pectin is the starting point that we propose for the various different functions because it can be directly/indirectly affected by three kinds of factors: promoting factors such as IAA, inhibitory factors such as ABA, and pectases; to induce pectin loosening, gel formation, and pectin degradation, respectively. These functions are closely related to complex cellular activities in the four zones of the root. In the meristematic zone, where tight junctions between cells are important for maintaining cell division and limited growth in the zone at the end of the primary meristem, pectin degradation should be under strict control, and an appropriate degree of gel formation is necessary. Therefore, an increased degree of pectin unmethylesterification in the *Ostsd2* mutants may cause more pectin degradation by pectase followed by cell separation and formation of schizogenous aerenchyma (Patterson, 2001; Rhee *et al.*, 2003). In the elongation zone, an appropriate degree of pectin unmethylesterification is necessary (Bosch and Hepler, 2005) in order to enable promoting factors such as IAA to initiate pectin loosening and cell extension, whereas binding of inhibitory factors such as ABA should be reduced in order to support cell extension in particular. Therefore, the increased degree of unmethylesterification may cause greater binding of inhibitors to form gels and reduced cell elongation in the *Ostsd2* mutants. In the mature zone, especially in the cells of the pericycle, pectin degradation (Laskowski *et al.*, 2006) or loosening is required, whereas gel formation is disadvantageous for cell division and lateral root formation; therefore, an increased degree of unmethylesterification may cause more pectin loosening and the formation of more lateral roots in the *Ostsd2* mutants. Of course, there is the fourth zone, the root cap; pectin’s role in forming normal border cells has been elucidated in Arabidopsis (Durand *et al.*, 2009), and this is logically consistent with our hypothesis presented here. This hypothesis elucidates the competitive relationships between promoting factors, inhibitory factors, and pectase in the regulation of pectin’s role in cell extension/division in a given zone. These relationships suggest that the established and subtle regulation of pectin is complicated, thus indicating, intriguingly, that pectin has multiple biological roles.

## Supplementary data

Supplementary data are available at *JXB* online.

Figure S1. Homologous sequence alignments between AtTSD2 and three proteins in rice.

Figure S2. Longitudinal sections of the root tips of WT and *Ostsd2* plants without or with ABA treatment.

Figure S3. Ratio of longitudinal to transverse axis of the elongation zone cells.

Figure S4. Transverse sections of WT, *Ostsd2b*, and *Ostsd2c* roots in the meristematic zone and elongation zone with or without exogenous ABA treatment.

Figure S5. Immunofluorescent detection of HG methylation patterns using the monoclonal antibody LM19 in the *Ostsd2b* line.

Figure S6. Relative transcription levels of marker genes involved in the ABA synthesis and signaling pathways.

Table S1. Primers used in this study.

Supplementary Data

## Abbreviations:

ABA: abscisic acid
HG: homogalacturonan
HG-MT: homogalacturonan methyltransferase
IAA: indole-3-acetic acid
PME: pectic methylesterase
TSD: tumorous shoot development.
