# Supplementary material for: Rice putative methyltransferase gene OsTSD2 is required for root development involving pectin modification
Source: J Exp Bot. 2016 Aug 6;67(18):5349–62. doi: 10.1093/jxb/erw297 (PMC5049386; doi:10.1093/jxb/erw297)
Supplement: Supplementary Data [file supp_67_18_5349__index.html]

Rice putative methyltransferase gene OsTSD2 is required for root development involving pectin modification — Rice putative methyltransferase gene OsTSD2 is required for root development involving pectin modification — Supplementary Data 

# Rice putative methyltransferase gene *OsTSD2* is required for root development involving pectin modification

## Supplementary Data

Data files

- supplementary\_figures\_legends.docx - Supplementary Data
- supplementary\_figure\_S1.tif - Supplementary Data
- supplementary\_figure\_S2.tif - Supplementary Data
- supplementary\_figure\_S3.tif - Supplementary Data
- supplementary\_figure\_S4.tif - Supplementary Data
- supplementary\_figure\_S5.tif - Supplementary Data
- supplementary\_figure\_S6.tif - Supplementary Data
- supplementary\_table\_S1.pdf - Supplementary Data
