# Supplementary material for: Rice putative methyltransferase gene OsTSD2 is required for root development involving pectin modification
Source: J Exp Bot. 2016 Aug 6;67(18):5349–62. doi: 10.1093/jxb/erw297 (PMC5049386; doi:10.1093/jxb/erw297)
Supplement: Supplementary Data [file supp_erw297_supplementary_figures_legends.docx]

Figure legends

Fig. S1. Homologous sequences alignment between AtTSD2 and three proteins in rice.

Fig. S2. Longitudinal sections of the root tips of WT and *Ostsd2* without or with ABA treatment.

Fig. S3. Ratio of longitudinal to transverse axis of the elongation zone cells.

Fig. S4. Transverse sections of WT, *Ostsd2b*, and *Ostsd2c* roots in the meristematic zone and elongation zone with or without exogenous ABA treatment.

Fig. S5. Immunofluorescent detection of HG methylation pattern by monoclonal antibody LM19 in *Ostsd2b* line.

Fig. S6. Relative transcription level of marker genes involved in ABA synthesis and signaling pathway.
