# Supplementary figures and images for: Rice putative methyltransferase gene OsTSD2 is required for root development involving pectin modification
Source: J Exp Bot. 2016 Aug 6;67(18):5349–62. doi: 10.1093/jxb/erw297 (PMC5049386; doi:10.1093/jxb/erw297)

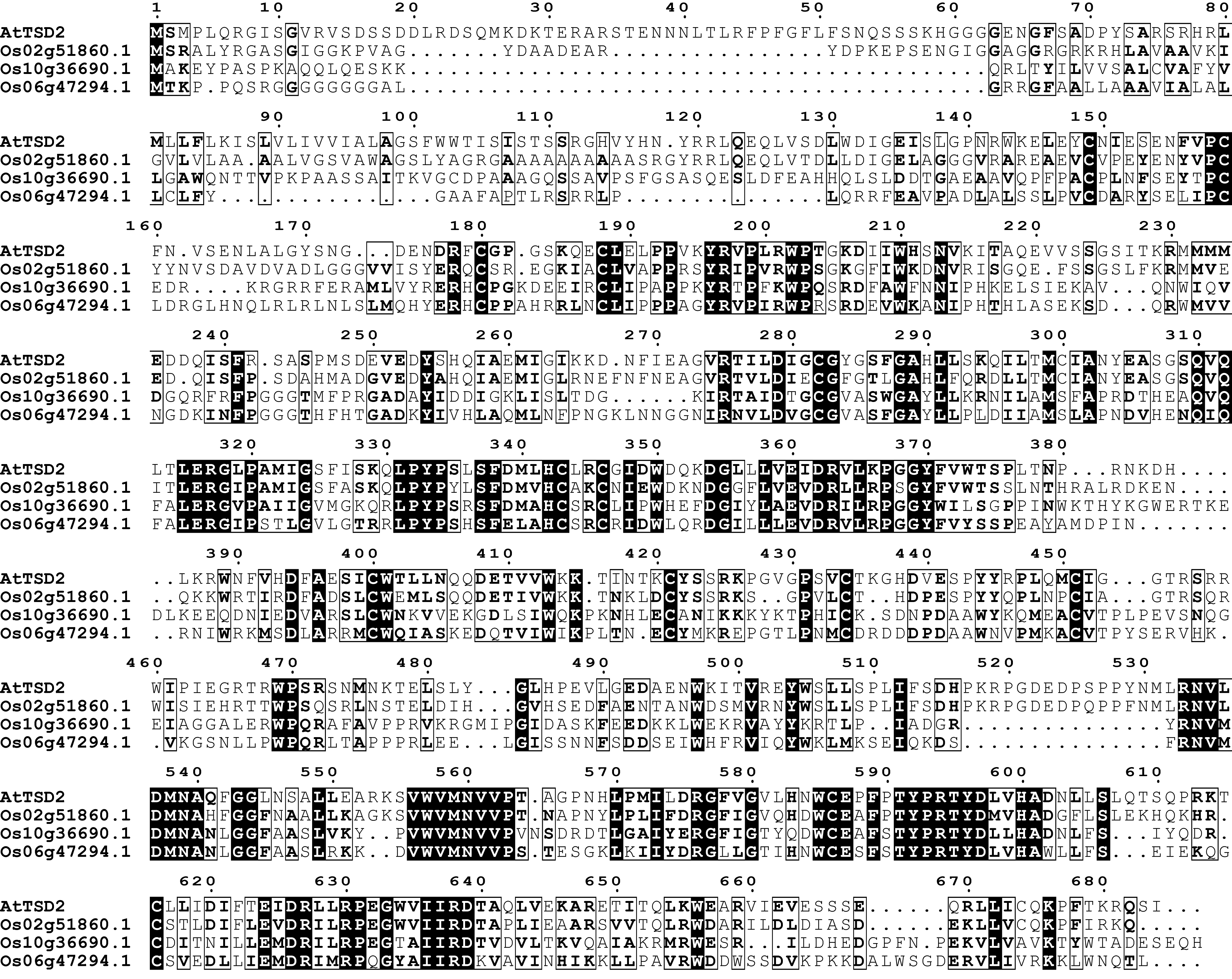

Supplement: Supplementary Data [file supp_erw297_supplementary_figure_S1.tif]

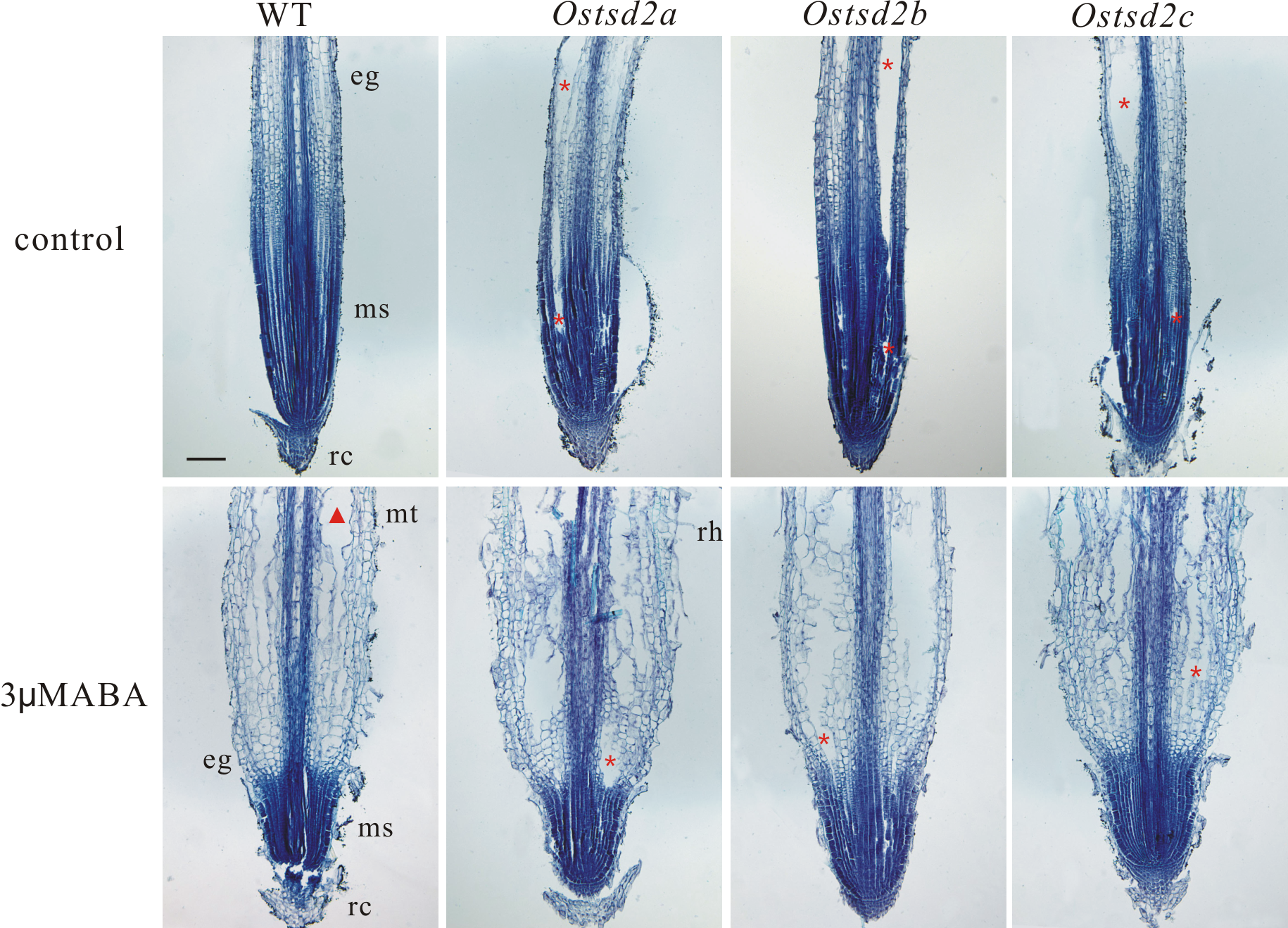

Supplement: Supplementary Data [file supp_erw297_supplementary_figure_S2.tif]

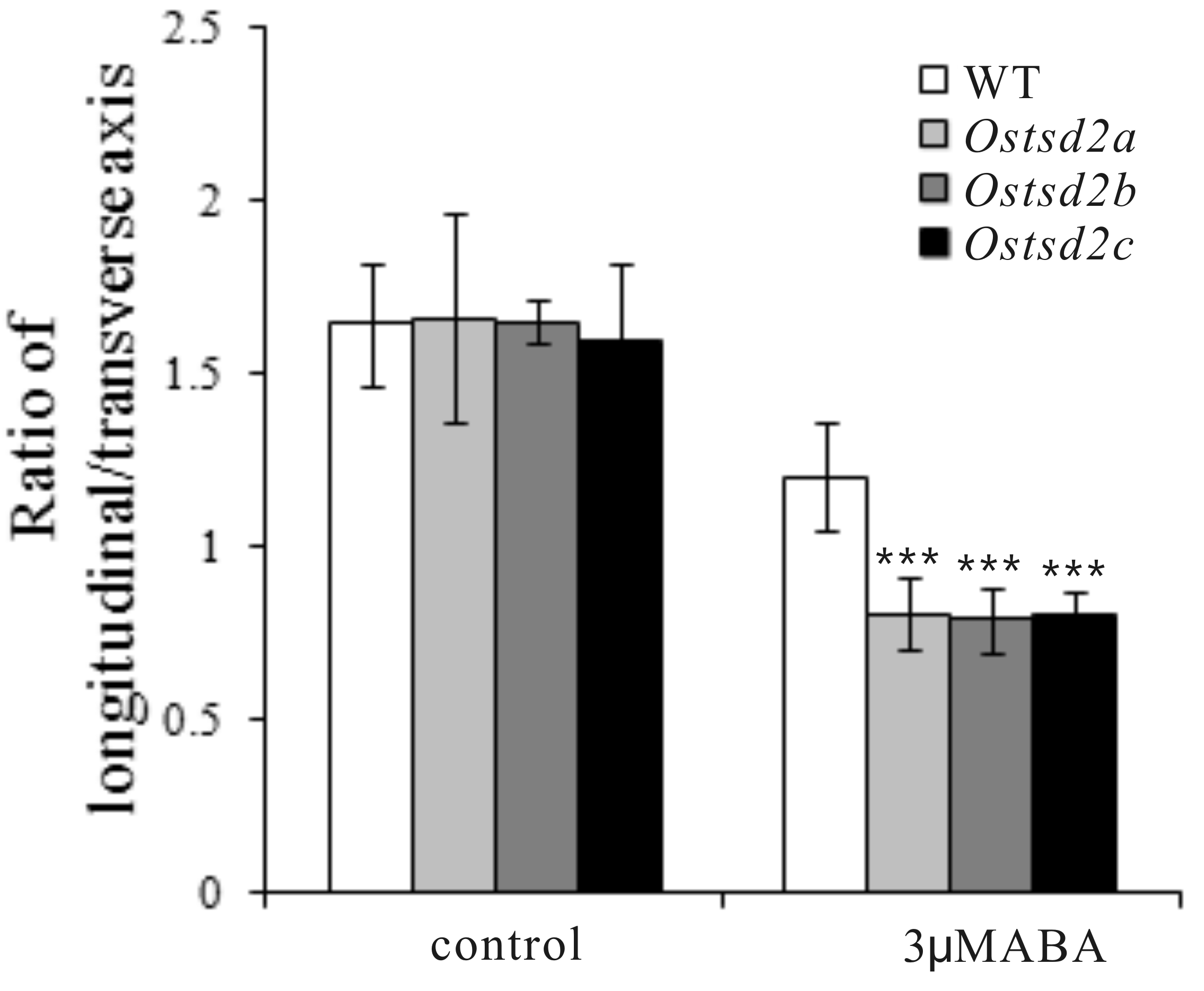

Supplement: Supplementary Data [file supp_erw297_supplementary_figure_S3.tif]

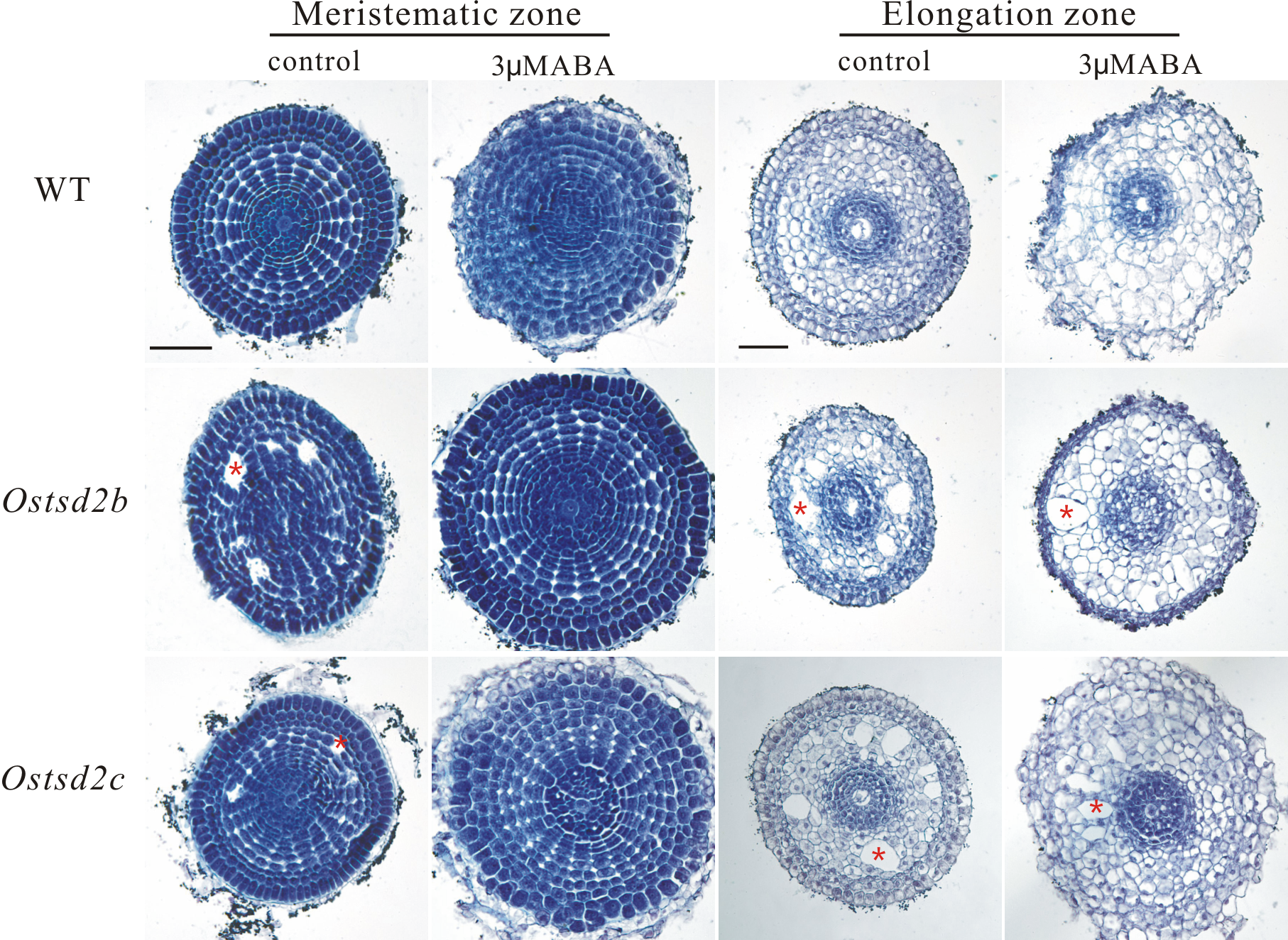

Supplement: Supplementary Data [file supp_erw297_supplementary_figure_S4.tif]

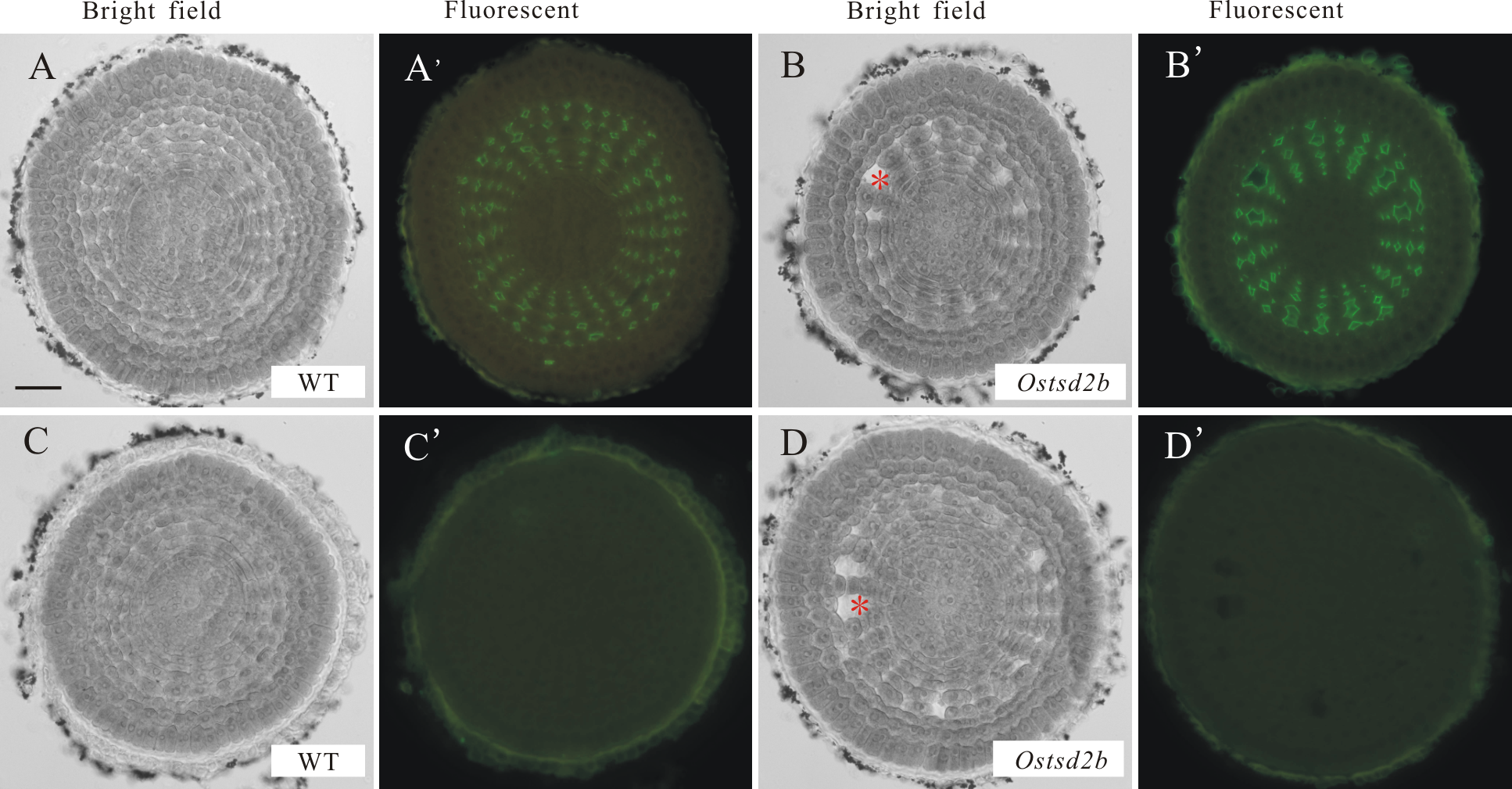

Supplement: Supplementary Data [file supp_erw297_supplementary_figure_S5.tif]

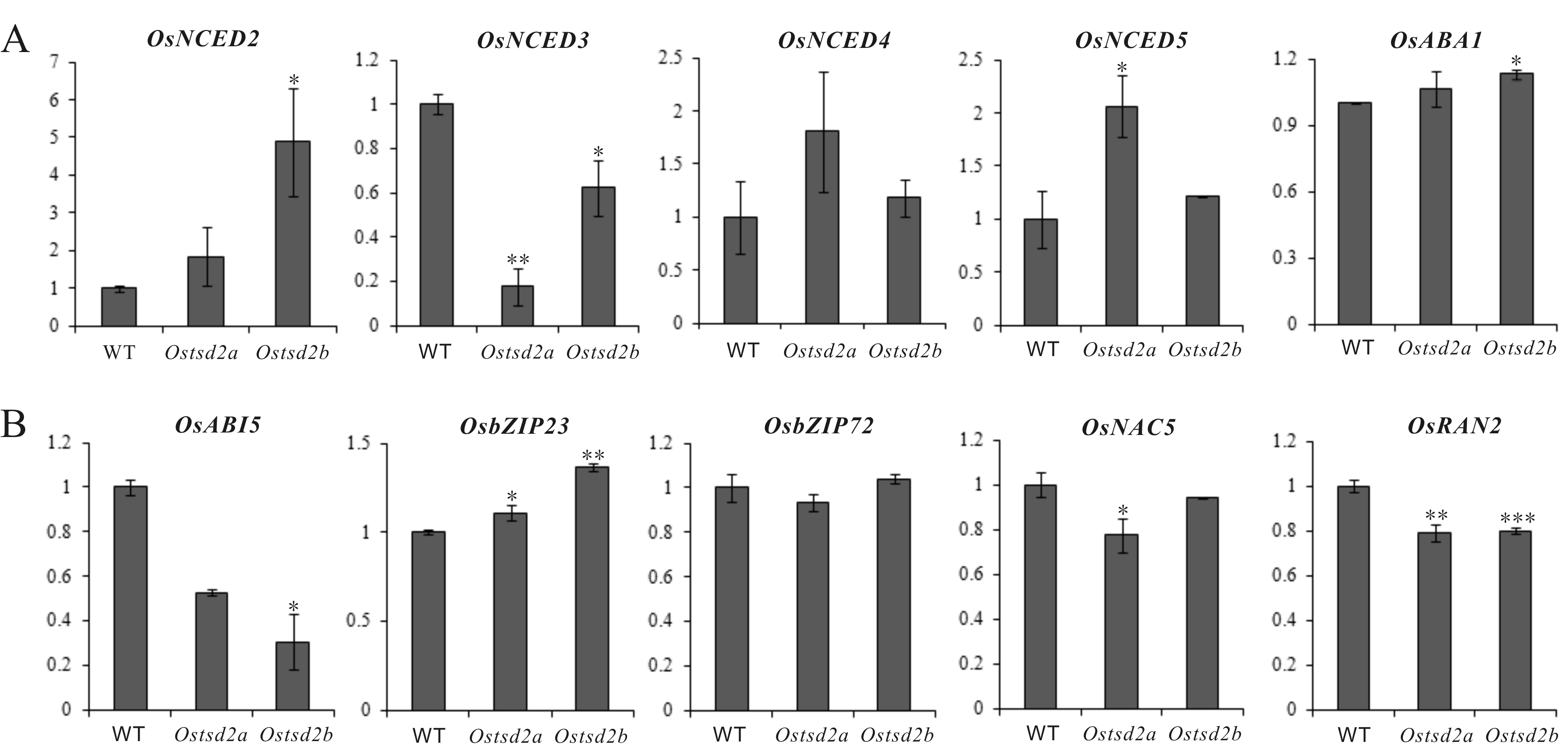

Supplement: Supplementary Data [file supp_erw297_supplementary_figure_S6.tif]
