# Supplementary material for: Rice putative methyltransferase gene OsTSD2 is required for root development involving pectin modification
Source: J Exp Bot. 2016 Aug 6;67(18):5349–62. doi: 10.1093/jxb/erw297 (PMC5049386; doi:10.1093/jxb/erw297)
Supplement: Supplementary Data [file supp_erw297_supplementary_table_S1.pdf]

Tab. S1. Primers used in this study.

Gene	Primers(5’-3’)	Purpose
OsTSD2	F: TGGAAGTTGATCGCATCCTAC<br>R: CCACCTGAGCTGTGTTACAA	Real-time PCR
OsNCED2	F:GGTATGGAAACGAGGATAGTGGTT<br>R: TGCTTATTGTTGTGCGAGAAGTTC	Real-time PCR
OsNCED3	F: CCCCTCCCAAACCATCCAAACCGA<br>R: TGTGAGCATATCCTGGCGTCGTGA	Real-time PCR
OsNCED4	F: TCCATCTCCTTCTCCCTCCTCCCA<br>R: CCTCGCACCCTGCTTGATCTTGCC	Real-time PCR
OsNCED5	F: ACATCCGAGCTCCTCGTCGTGAA<br>R: TTGGAAGGTGTTTTGGAATGAACCA	Real-time PCR
OsABA1	F: GAGTTGGTGGGAGATTCTTCAT<br>R: CAGCTTAACGGTCTTCCTTCT	Real-time PCR
OsABI5	F: CTCGTCTCAAAGAGGCAGAGAAG<br>R: TCTTCTCCTTGGACTGCTCCATC	Real-time PCR
OsbZIP23	F: GGAGCAGCAAAAGAATGAGG<br>R: GGTCTTCAGCTTCACCATCC	Real-time PCR
OsbZIP72	F: CCATTTGGAAGAAGGAAGAGACTT<br>R: CCATCGACCACTCGTCATCA	Real-time PCR
OsNAC5	F: TTAACGGAACGTGACGCCTCTG<br>R: ACGATCGACCTGACAGTGGTTAG	Real-time PCR
OsRAN2	F: ATTTCGTTGAAGCTGTTGC<br>R: CAAGCTCAGCCTCATGCT	Real-time PCR
OsUBI	F: GAGCCCAGCCCTTCAGATTC<br>R: TGGCATACAGGCGAGTCCAC	Real-time PCR
OsTSD2	F: AAAAAGCAGGCTTACAGGGAAATCTCGGGTTGTC<br>R: AGAAAGCTGGGTACGCTGCGGATCTCACGGC	Vector construction
